# Percutaneous contrast-enhanced ultrasound for localization and diagnosis of sentinel lymph node in early breast cancer

**DOI:** 10.1038/s41598-019-49736-3

**Published:** 2019-09-19

**Authors:** Jian Liu, Xiaoling Liu, Jiao He, Bo Gou, Yujie Luo, Sihui Deng, Hong Wen, Lin Zhou

**Affiliations:** 1 Department of Ultrasound, The First Affiliated Hospital of Chengdu Medical College, Chengdu, Sichuan 610500 China; 2 Department of Ultrasound, The Affiliated Hospital of North Sichuan Medical College, Nanchong, Sichuan 637000 China

**Keywords:** Breast cancer, Breast cancer

## Abstract

This study assessed the efficacy of percutaneous contrast-enhanced ultrasound (CEUS) in localization sentinel lymph node (SLNs) for biopsy and diagnosis of metastatic SLNs in patients with early breast cancer. From January to November 2017, seventy-five patients with early breast cancer confirmed by pathology were enrolled in this study. CEUS was performed after subdermal injection of ultrasound contrast agent (SonoVue, 2.0 ml in total dose) around the areola on the ipsilateral side of the breast. The contrast-enhanced lymphatic vessels and associated SLNs were observed and traced in real time. The lymphatic vessels and SLN were mapped and labeled on the skin surface. Sentinel lymph node biopsy (SLNB) was performed after injection of 2.0 ml methylene blue at same injection site of SonoVue. The accuracy of percutaneous CEUS localization of SLNs was determined compared to blue dye injection technique. The pathological results under blue dye guided biopsy were used as the reference standard to calculate the sensitivity and specificity of CEUS for the diagnosis of SLNs. A total of 163 SLNs obtained through SLNB following methylene blue tracing in 75 patients. There were 116 SLNs identified by percutaneous CEUS. The difference of detection rates between blue dye and CEUS was statistically significant (Z = −2.651, P = 0.008). The identification rate of SLNs by CEUS was 71.17% (116/163). The accuracy of percutaneous CEUS localization of axillary SLNs was 94.67% (71/75) compared to blue dye-guided biopsy. Among the 116 SLNs detected by percutaneous CEUS, pathologic results showed 51 positive SLNs and 65 negative SLNs whiles CEUS findings indicated 83 positive SLNs and 33 negative SLNs. Only 50 of 83 SLNs had metastasis on pathology, while 33 were detected as false positive. The sensitivity and specificity of CEUS for the diagnosis of metastatic SLN was 98.04%(50/51) and 49.23%(32/65), respectively. Percutaneous CEUS can be used as an effective method to localize the SLNs for guiding SLNB. This method has excellent sensitivity for identifying the SLNs but lower specificity for detecting metastatic SLNs in patients with early stage breast cancer.

## Introduction

Breast cancer is the second most commonly diagnosed cancer among American women. Currently, the average risk of a woman in the United States developing breast cancer sometime in her life is about 12%. An estimated 268,600 new cases of invasive breast cancer will be diagnosed in women in the United States in 2019, according to the American Cancer Society^1^. Pretreatment staging of breast cancer become critical for clinical management. Whether axillary lymph node metastasis is an important factor affecting the prognosis of breast cancer patients^2^. Sentinel lymph node biopsy (SLNB) has gradually replaced traditional axillary lymph node dissection (ALND) as the standard of care in early breast cancers. Less invasive SLNB represents a highly accurate and less-morbid axillary staging, which allows most patients to avoid unnecessary ALND^3^.

The sentinel lymph node (SLNs) is the hypothetical first lymph node or group of nodes draining a local tissue or primary cancer. The status of SLNs is very important since lymph node metastasis is one of the most critical prognostic factors. In order to make appropriate strategy for treatment, CT, MRI, and PET are applied for the evaluation and localization of regional lymph nodes in patients with breast cancer. Although these imaging techniques provide useful information for surgical and non-surgical treatments, the sensitivity and specificity for the diagnosis of lymph node status could not be compatible to or replace SLNB^4^. Clinically. lymphoscintigraphy and blue dye mapping are commonly used to identify the SLNs during surgical treatment of breast cancer. In recent years, contrast-enhanced ultrasound (CEUS) using microbubble-based agents has investigated for evaluation of lymphatic system in both animal and human studies^5,6^. The aim of this study was to investigate the efficacy of percutaneous CEUS in localization SLNs for guiding the biopsy and diagnosis of metastatic SLNs using blue dye-guided pathology as reference standard in patients with early breast cancer..

## Materials and Methods

### Patients

This study was approved by the medical ethics committee of the first affiliated hospital of chengdu medical college. The methods were carried out in accordance with the approved guidelines and written informed consents were obtained from all participants prior to enrollment. From January to November 2017, consecutive patients with early breast cancer admitted to our hospital were enrolled in this study. The inclusion criteria were: (1) breast cancer confirmed by needle biopsy or local excision; (2) preoperative clinical staged as Tis, T1N0M0 or T2N0M0 breast cancer; (3) no palpable axillary lymph nodes; and (4) qualified candidates for SLNB. Exclusion criteria were: (1) history of axillary surgery; (2) pregnancy or lactation; and (3) allergic to ultrasound contrast agents. Finally, 75 patients were enrolled in this study, including 74 females and 1 male, mean age of 49.3 ± 8.4 years from 31 to 71 years. Among them, there were 58 invasive ductal carcinomas, 9 ductal carcinomas *in situ*, 7 invasive lobular carcinomas, and 1 mucinous carcinoma. All patients underwent routine ultrasonography and percutaneous CEUS for evaluation of SLNs 1 day before SLNB procedure.

### Instruments and methods

Ultrasound examination was performed using EPIC Q7 (Philips ultrasound system, Holland) equipped with high-frequency linear array broadband matrix probes with frequency of 7–12 MHz and contrast pulse sequence(CPS) imaging technology. Low mechanical index (MI) values were applied (MI: 0.06) to reduce microbubble destruction. The ultrasound contrast agent used in this study was microbubbles (Sonovue, Bracco Imaging, Milan, Italy). Sonovue dry powder was mixed with 2 ml sterile saline.

Patient was placed in supine position with exposure of the affected breast and axillary area. Grey-scale and color Doppler ultrasound was used to evaluate the breast lesion and the axillary lymph nodes. The location, size, shape, echotexture, and internal blood flow of the breast lesion and lymph nodes were digitally recorded on the hard drive of the unit for later analysis.

**Figure 1:**
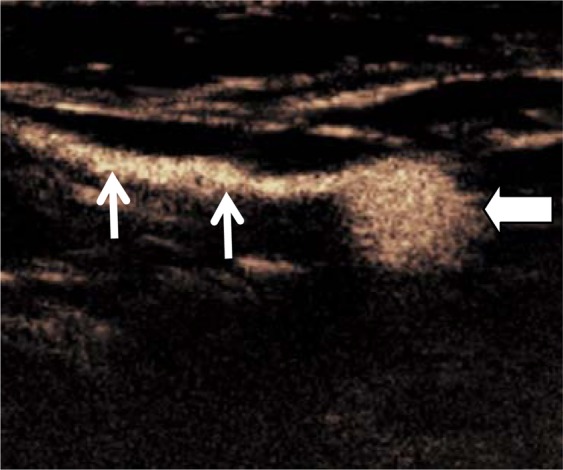
Percutaneous CEUS identified a SLN (bold arrow) along with enhanced lymphatic vessel (thin arrows).

Percutaneous CEUS was conducted for identifying lymphatic vessels and axillary lymph nodes on the lesion-side breast. Subcutaneous local infiltration anesthesia was performed with 2% lidocaine at 12, 3, 6 and 9 o’clock around the areola. A 0.5 ml Sonovue suspension was intradermally injected at each anesthesia point (total of 2.0 ml) and the injection area was gently massaged for 5s–10s in order to promote the drainage of contrast agent to the lymphatic vessels. Subcutaneous lymphatic channels could be visualised immediately on CPS after injection. Enhanced lymph nodes could be detected by moving the probe along the channels. The first or first group of enhanced lymph nodes were considered as SLNs (Fig. 1) along the enhanced lymphatic vessels. Live dual images were used to confirm the presence of an architecturally defined SLN. If the lymphatic channel or lymph node was not detected successfully, one or two additional injections could be performed. Once identified, lymphatic duct and SLNs were marked on the skin surface. This serves as a road map so that the SLNs can be identified easily by surgeons for SLNB purpose. The size, number, location, distance to the body surface and enhancement appearances of the SLNs were documented. The correspondent location of the SLNs was marked on the body surface for SLNB purpose.

**Figure 2:**
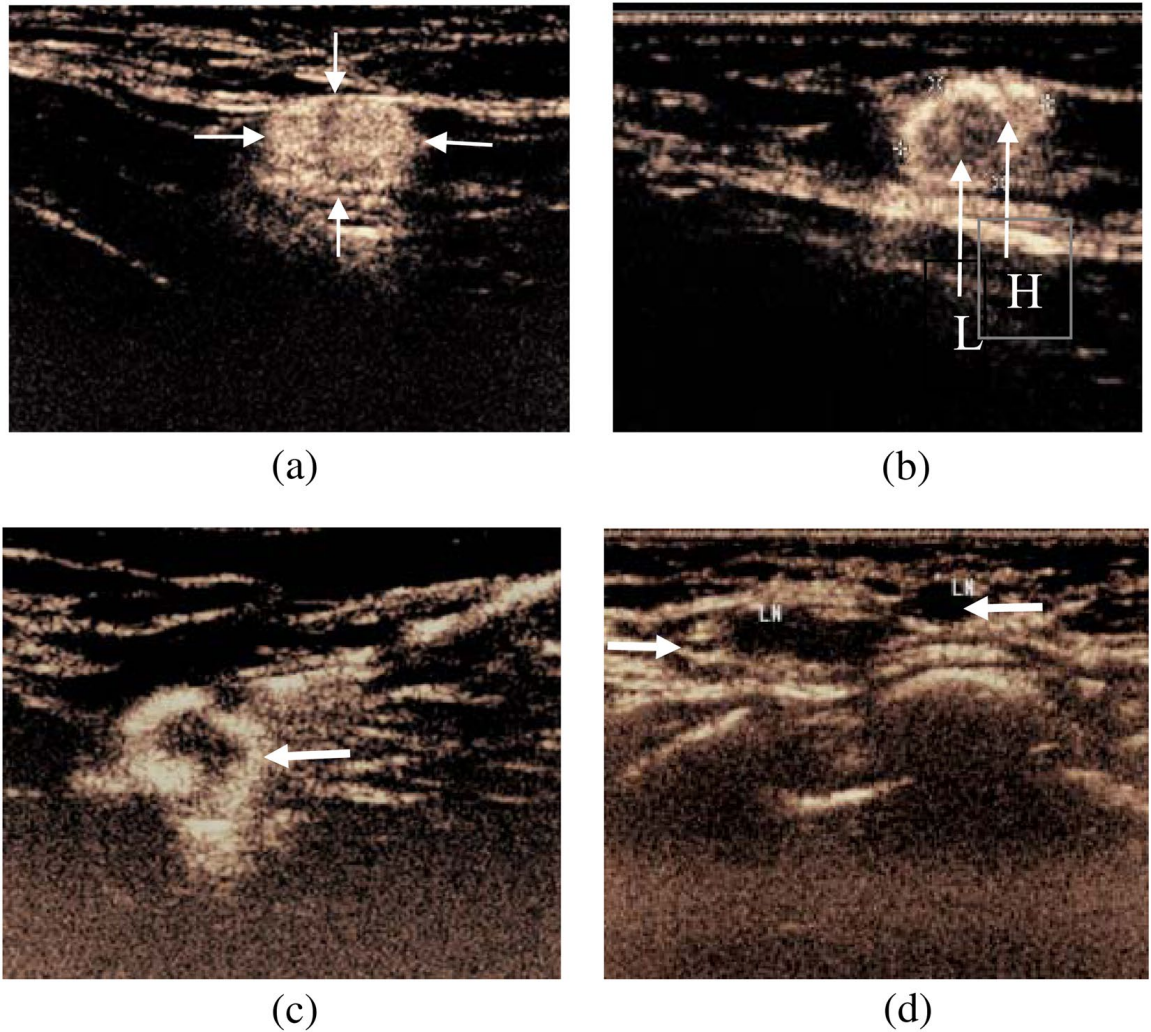
CEUS enhancement of SLNs in 4 types. (**a**) Overall uniform high enhancement (arrow); (**b**) Uneven enhancement with mixture of high (H) and low (L) enhancement(arrow); (**c**) Peripheral annular high enhancement, low or no enhancement inside(arrow); and (**d**) No enhancement link with lymphatic vessel (arrow).

According to different perfusion features of SLNs^7,8^, the enhancement was divided into the following four types: Type 1: overall uniform enhancement (Fig. 2a); Type 2: uneven enhancement with mixture of high and low enhancement (Fig. 2b); Type 3: peripheral complete or incomplete annular enhancement with low or no center enhancement (Fig. 2c); Type 4: no enhancement or weak enhancement of the node connected with lymphatic vessel (Fig. 2d). Among them, the type 1 of SLNs was considered as negative nodes (no metastasis), while the other three types of SLNs were considered as positive nodes (metastasis).

### SLNB and pathological evaluation

Immediately after anaesthesia induction, Methylene blue (Jumpcan Pharmaceuticals, China) was intradermally injected at 12, 3, 6 and 9 o’clock around the areola at dose of 0.5 ml for each point (total 2.0 ml) 10 min before SLNB procedure. Under ultrasound guidance and skin mark as reference, a 4-cm incision in the armpit was made to looking for blue-stained lymphatic vessels on the outer edge of the pectoralis major muscle, and also detecting blue-stained lymph nodes along with lymphatic vessels. The blue-stained lymph nodes or blue-stained lymphatic vessels leading to lymph nodes were considered as SLNs, which would be resected for pathological analysis. Intraoperative cytological examination and postoperative HE staining were conducted on these resected lymph nodes. Microscopic inspection was used to confirm the tumor metastases as positive SLN, and vice versa for negative SLNs.

### Statistical analysis

In this study, commercially available statistical software (SPSS19.0, SPSS Inc. Chicago, IL, USA) was used for data analysis. The quantitative data were expressed as $\overline{x}$ ± *s*, and the difference of SLNs number detected by percutaneous CEUS and SLNB was compared by Wilcoxon rank sum test. In comparison with the surgical results, the accuracy of percutaneous CEUS localization of SLN was calculated, and the pathological results were used as the reference standard to calculate the sensitivity and specificity of CEUS for the diagnosis of SLN metastasis. *P* < 0.05 was regarded as being statistically significant.

## Results

There were no adverse effects and complications in any patients after receiving percutaneous CEUS. The locations of breast lesions included 36 in the left side and 39 in the right side with 33 in the outer upper quadrant, 17 in the inside upper quadrant, 9 in the inside lower quadrant, 11 in the outer lower quadrant, and 5 in the areola area. The largest diameter of the mass ranged from 0.88 to 5.72 cm with average size of 2.87 ± 1.09 cm.

Under CEUS and blue dye-straining guidance, 163 SLNs in 75 patients were obtained from SLNB, with an average SLN numbers of 2.42 ± 0.93 per case. The lymphatic vessels in all 75 patients were able to identify by CEUS. Among them, enhanced SLNs were identified in 62 patients and not visualized in 13 patients. Of the 62 cases, 116 SLNs were detected by CEUS with an average SLNs of 1.50 ± 0.92 per case. Blue dye-guided SLNB obtained additional 47 SLNs compared to percutaneous CEUS, and the difference was statistically significant (Z = −2.651, P = 0.008). The identification rate of SLNs by CEUS was 71.17% (116/163).

Of the 62 cases, lymphatic vessels and 116 SLNs detected by CEUS were located on the body surface. This 116 SLNs were detected by blue dye and were successfully removed by SLNE. In 13 of 75 patients, CEUS failed to observe SLN, which were located by conventional ultrasound and enhanced lymphatic vessels direction. As a result, 9 cases were successfully removed in SLNB, and 4 cases failed to locate by CEUS. The accuracy of percutaneous CEUS localization of axillary SLNs was 94.67% (71/75) compared to blue dye-guided biopsy.

Among the 116 SLNs detected by percutaneous CEUS, there were 83 SLNs having types 2, 3, 4 of enhancement patterns, i.e., uneven enhancement, ring enhancement or no enhancement, which were considered as positive SLN by definition. By comparing the CEUS diagnosis with the pathological diagnosis of SLNs, pathologic results showed 51 positive SLNs and 65 negative SLNs while CEUS findings indicated 83 positive SLNs and 33 negative SLNs. Only 50 of 83 SLNs had metastasis on pathology, while 33 were detected as false positive, in which pathological findings showed lymphoid follicle hyperplasia and lymphatic sinus dilatation (n = 12), lymph node reactive hyperplasia (=6), adipose tissue deposition (n = 8), fibrotic and lymphoid tissue deposition (n = 5), and lymphatic vessels with cancerous plugs (n = 2). The sensitivity and specificity of CEUS for the diagnosis of SLN was 98.04% (50/51) and 49.23% (32/65), respectively. The pathological results of 116 SLNs and the CEUS enhancement types were shown in Table 1.

**Table 1:** Pathological results of 116 SLNs and CEUS enhancement types.

Pathology	Enhancement types of SLNs				Total
	Type 1	Type 2	Type 3	Type 4	
Positive SLN	1	29	10	11	51
Negative SLN	32	24	6	3	65
Sub total	33	53	16	14	116

## Discussion

In clinical setting, two methods for detection of SLNs have been used either independently or in combination: (1) injection of blue dye with surgical dissection providing visual identification of draining LVs as well as SLNs; (2) injection of radioisotopes followed by evaluation with a gamma camera (scintigraphy) or intraoperatively with a gamma probe (isotope mapping)^9,10^. In general, blue dye and isotope detection of SLNs are accurate procedures, but pitfalls do exist^11^. There is wide variation in the sensitivity for the detection of SLNs ranging from as low as 76% to as high as 97%^12^. Blue dye mapping is more often used to localize SLNs of breast cancer for guidance of SLNB, while the application of radionuclide is limited in SLNB due to radioactive pollution and its legislative issues^11^.

This study was to investigate the accuracy of percutaneous CEUS in the localization of SLNs to guide SLNB in patients with early breast cancer. When compared with methylene blue staining method as reference standard, the accuracy of CEUS localization was 71.17% (116/ 163), consistent with previous literature reports^13^.

The blue dye injection is able to identify blue-stained lymphatic vessels and SLNs^14^, which is easy to visualize the color adjacent to adipose tissue within the nodes. However, if the dye permeates into non-SLN tissue, it may create a false positive. Our study demonstrated that localization of SLNs by percutaneous CEUS could be used as an effective supplement to SLNB procedure, reducing false positive rates. Among the 75 patients in this study, 116 SLNs were detected by percutaneous CEUS, while163 SLNB were found by methylene blue mapping. There is statistically significant difference (P = 0.008) between CEUS and Blue dye methods. Thus, further investigation to improve the SLN detection rate is still needed.

Previous studies^15–19^ revealed that percutaneous CEUS is valuable technique for evaluation of SLN in breast cancer because it was non-invasive, non-radiative, easy to use and cost effective. In this study, we demonstrated that real time CEUS is able to observe enhanced lymphatic vessels and SLNs as well as classify enhancement patterns of SLNs. The pathological results were used as the gold standard to confirm the value of CEUS in determining SLN involvement. As results, there are four enhancement patterns of SLNs seen: (1) uniform enhancement of entire node, suggesting no metastasis in the node, and the contrast agent spreads evenly within the lymph node. (2) uneven enhancement and variable intensity within the node, which may result from tumor infiltrating and forming a partial filling defect in the node. (3) peripheral complete or incomplete annular enhancement, with low or no center enhancement, suggesting tumor invading peripheral region under the capsule and then destroying the para-sacral sinus and sinus of the node. The tumor could form in the parenchyma, mixed with the surrounding lymphoid tissue, and inducing neovascularization. Therefore, the ring-shaped enhancement from the periphery, uneven distribution in the parenchyma, and low-or-no perfusion area could represent the metastasis node^20^; (4) no or low enhancement of the node associated with enhanced lymphatic vessel, suggesting metastatic lymph nodes. Tumor cells may infiltrate most lymph nodes replacing all normal tissue, or blockage of main lymphatic vessels, resulting in the failure of the contrast agent filling. Then, SLN shows weak enhancement or no enhancement^21^.

The results of this study showed that the sensitivity and specificity of CEUS for the diagnosis of SLN metastasis was 98.04% (50/51) and 49.23% (32/65), respectively. The sensitivity was high but the specificity was rather low. There were 83 SLN having types 2, 3, 4 of enhancement, which were considered as positive SLN by classification. Only 50 of 83 SLNs were having metastasis while 33 were no tumor detected as false positive. The reasons for the high false positives could be as follows: (1) SLNs in early breast cancer may have lymphoid follicle hyperplasia, lymphatic sinus expansion and reactive hyperplasia. Although previous studies have shown that percutaneous CEUS could effectively delineate lymphatic vessels and SLNs, and the tumor infiltration would be considered when there was no enhancement or uneven enhancement of the lymph nodes^5,7^. However, benign lesions such as lymphoid follicle hyperplasia, lymphatic sinus dilation and chronic inflammation could also block lymphatic drainage, resulting in retention of contrast media in the lymphoid parenchyma, which was characterized by uneven enhancement^22^. (2) There are 8 SLNs in this group showing uneven enhancement and suspected metastases. However, the pathological results demonstrated adipose tissue within the nodes. This could be due to fat infiltration or its wrapping around SLN, resulting in uneven enhancement of SLN and false positive. (3) There were additional 2 SLNs with fibrotic tissue infiltration showing no enhancement in CEUS. Postoperative pathology confirmed that the tumor thrombus blocked the lymphatic vessels and the contrast agent could not enter into the nodes and no tumor found in the SLNs.

In summary, percutaneous CEUS could effectively localize SLNs for guiding SLNB in patients with early breast cancer, and also could be used to evaluate its tumor involvement based on enhancement patterns of the nodes. However, specificity of diagnosis of SLN metastasis is low. The SLNs with unevenly enhanced or not enhanced have to be cautiously considered as positive nodes. The enhanced patterns for characteristics of SLN need further study to improve specificity and provide more valuable information for SLN staging.
